# Quantitative Analysis of SARS-CoV-2 Serological Responses Post Three Doses of Immunization and Prior to Breakthrough COVID-19 Infections

**DOI:** 10.3390/vaccines10101590

**Published:** 2022-09-22

**Authors:** Kathryn Macrae, Catherine Yuqing Gong, Prameet Sheth, Jorge Martinez-Cajas, Yanping Gong

**Affiliations:** 1 Department of Pathology and Molecular Medicine, Kingston Health Sciences Centre, Queen’s University, Kingston, ON K7L 3N6, Canada; 2 Division of Infectious Diseases, Kingston Health Sciences Centre, Queen’s University, Kingston, ON K7L 3N6, Canada

**Keywords:** SARS-CoV-2, vaccine, immunity, serology

## Abstract

Background: Vaccine mediated SARS-CoV-2 antibody responses should be carefully evaluated. With regular follow-up in healthy individuals, we aimed to determine SARS-CoV-2 serological responses post three doses of immunization and prior to breakthrough infections in the Canadian population. Methods: In a prospective cohort study, we enrolled 140 healthy participants post COVID-19 vaccination in Kingston, Ontario, Canada. IgG antibodies against the SARS-CoV-2 spike receptor–binding domain were quantified by immunoassay post three doses of immunization. With COVID-19 rapid antigen test, polymerase chain reaction, and whole genome sequencing, 27 breakthrough infections were identified. Results: Following SARS-CoV-2 vaccine (including BNT162b2, AZD1222, and mRNA-1273), the median serum anti-spike protein antibody level was 143.6 BAU/mL (binding antibody unit, interquartile range 79.0–266.6) post the first dose of immunization, 1046.4 BAU/mL (423.9–1738.2) post the second dose, and 1604.7 BAU/mL (700.1–3764.0) post the third dose. Observed differences were significant (*p* ≤ 0.001). The median antibody level of 1604.7 BAU/mL post third dose is 45.6 times that of the seroconversion level (35.2 BAU/mL). This indicates that most vaccines approved are effective in producing robust antibody responses. In seven breakthrough cases characterized by whole genome sequencing, prior to infection, antibody concentrations of breakthrough cases were at 3249.4 (Delta), 2748.4 (Delta), 4893.9 (Omicron), 209.1 (Omicron), and 231.5 (Omicron), 725.7 (Omicron), and 2346.6 (Omicron) BAU/mL. Compared with the average antibody concentration of 2057.7 BAU/mL (58 times that of the seroconversion concentration) from above seven cases, 37.2% of triple vaccinated, 19.0% of double vaccinated, and 1.5% single dosed individuals have higher SARS-CoV-2 antibody levels. Conclusions: Most vaccines are effective in producing robust antibody responses when more than one dose is given, and the more doses the higher the serological response. Likely due to the highly contagious nature of SARS-CoV-2 variants, a significant number of participants have SARS-CoV-2 antibody responses lower than the average antibody concentration prior to the known breakthrough infections. Additional vaccination is likely required to ensure immunity against infection by SARS-CoV-2.

## 1. Introduction

It has been more than two years into the global pandemic of SARS-CoV-2 infection and over twelve billion doses of vaccines have been administered [1]. COVID-19 vaccine effectiveness should be carefully evaluated and explicitly defined, especially for mRNA vaccines which are based on new technology. Currently, Health Canada has approved six vaccines for a national immunization program, e.g., Moderna SpikeVax (mRNA, mRNA-1273), Pfizer-BioNTech Comirnaty (mRNA, BNT162b2), AstraZeneca Vaxzevria (viral vector-based, AZD1222), Janssen (Johnson & Johnson, New Brunswick, NJ, USA, viral-vector based, Ad26.COV2.S), Novavax Nuvaxovid (protein-based vaccine), Medicago Covifenz (plant based virus-like particle) [2]. The U.S. Food and Drug Administration (FDA) has approved similar COVID-19 vaccines for Emergency Use [3].

Based on the extensive knowledge from other vaccination programs, there are multiple markers to evaluate vaccine efficacy. These markers include antibody levels determined by enzyme-linked immunosorbent assay (ELISA), viral and bacterial neutralization assay, interferon assay, and hemagglutination assay [4]. ELISA is the most commonly used methodology to evaluate immunity after immunization. The ELISA based methodology generally outperforms immunochromatographic (ICT) assay for the detection of SARS-CoV-2 antibodies due to superior analytical sensitivity and specificity [5]. For most other vaccines, a universal cut-off based on semi-quantitative or quantitative ELISA is often chosen to represent protection and immunity [4]. As demonstrated by the Rubella vaccine, the cut-off value should be continuously monitored and adjusted with the aid of large epidemiological studies [6,7]. Due to our limited knowledge regarding the serological responses prior to breakthrough infection, it is unknown if a similar cut-off level for prevention against infection could be selected for SARS-CoV-2 vaccines.

Limited data exist about serological responses longitudinally post three doses of vaccination, as well as antibody levels prior to breakthrough COVID-19 infections. In this prospective study, we followed up immunized healthy individuals for antibody responses post three doses and prior to breakthrough infections. This knowledge is critical to evaluate serological responses and to determine the association between antibody levels and infection.

## 2. Materials and Methods

### 2.1. Recruitment, Sample, and Data Collection 

Institutional ethics committee approval and consent from participants were obtained. In this prospective cohort study from May 2021 to July 2022, we enrolled healthy participants post COVID-19 vaccination in Kingston, Ontario, Canada. The health status of participants was determined by volunteer reporting, and participants with underlying medical conditions potentially affecting their immune function were excluded in this study. 140 healthy participants were followed-up longitudinally. The interval between blood collection and a specific dose was predetermined with the intention of using one single blood collection to represent the likely antibody level before the next dose was offered. Participants were categorised into each group, based on the type of vaccine they received for their first, second, and third doses. If a participant received only BNT162b2 for their first, second, and third doses they were placed in the BNT162b2 category. If a participant received only AZD1222 for their first, second, and third doses they were placed in the AZD1222 category. If a participant received only mRNA-1273 for their first, second, and third doses they were placed in the mRNA-1273 category. If a participant received more than one of the following BNT162b2, AZD1222 or mRNA-1273 for their first, second, and third doses they were placed in the Mixed Dose category.

### 2.2. Quantitative Antibody Measurement

IgG antibodies against the SARS-CoV-2 spike receptor–binding domain were quantified by ELISA (EUROIMMUN, product number: EI 2606-9601-10). ELISA were coated with the spike protein in the receptor–binding domain of SARS-CoV-2 expressed recombinantly in the human cell line HEK 293. The method has been authorized for clinical use by Health Canada and Emergency Use Authorization by the FDA. This quantitative method has a linear range between 3.2 to 384 BAU/mL (binding antibody unit). Samples with results over 384 BAU/mL were diluted by a factor of 20 to 30-fold to obtain numeric results. A cut-off of 35.2 BAU/mL was used to determine the seroconversion (recommended by the method manufacturer).

### 2.3. Breakthrough Case Identification and Characterization

The infection status of the study participants was monitored by standard public health protocol in Ontario, Canada. Testing strategy using COVID-19 rapid antigen test and polymerase chain reaction (PCR) test was based on public health testing protocol. COVID-19 antigen rapid test (BTNX, Markham, ON, Canada) was distributed to participants by Queens’ University. PCR/Gene mutation analysis was performed at Kingston Public Health laboratory following standard protocol. Both positive and negative PCR results were reported to the Public Health Ontario database. To determine the variants of infection, PCR positive samples were further tested by whole genome sequencing (WGS) using the COVID Seq Test (Illumina). Libraries were loaded at 9 pM for 2 × 150 bp sequencing on the MiSeq instrument (Illumina). Sequencing files were de-multiplexed using the native instrument software for downstream analytics. Illumina’s iVar software was used to trim primer sequences and generate a consensus sequence with a minimum read depth of 50 and a minimum frequency threshold of 0.6. Phylogenies were created using Augur from Nextstrain’s bioinformatic toolkit (https://github.com/nextstrain/augur, accessed on 20 August 2022) using the ancestral genome (MN908947.3) as the reference and root for the phylogenetic trees.

### 2.4. Statistical Analysis

All statistical analysis was performed using R Statistical Software (the R Foundation, United State). A Shapiro–Wilk normality test was performed on each group to determine if it was normally distributed. Groups that were normally distributed had results reported as means. For groups that were not normally distributed, results were reported as medians and interquartile range (IQR), and a non-parametric Kruskal- Wallis test was performed to determine statistical significance amongst the groups.

## 3. Results

### 3.1. Characteristics of the Study Cohort

The baseline characteristics of study participants are summarized in Table 1. All 140 participants received SARS-CoV-2 vaccines following the recommended dose and dosing interval in Ontario, Canada. The median antibody concentrations were 143.6, 1046.4, and 1604.7 BAU/mL following the first, second, and third dose of the vaccine, respectively. On average, there was 7.2 times increase in antibody concentration from the first to second dose. From the second to third dose, there was an average increase of 1.5 times in antibody concentration. A Kruskal–Wallis test was conducted comparing the median antibody concentration between the three doses, which found a significant difference in these values (*p* < 0.001).

### 3.2. Characteristics of Breakthrough Cases

Table 2 describes the characteristics of seven breakthrough cases characterized by WGS with the variants identified. All breakthrough infections occurred post the second and prior to the third dose. The highest first dose antibody result was 538.2 BAU/mL, whereas the lowest was 118.1 BAU/mL. It is also interesting to note that the antibody levels vary significantly amongst the second dose antibody results prior to the breakthrough infections. The highest level of antibody generated after the second dose was 4893.9 BAU/mL, whereas the lowest level was 209.1 BAU/mL, with the mean concentration at 2057.7 BAU/mL. The third dose antibody concentrations, which were collected after infection and one booster dose, are also shown. The lowest antibody concentration was 5962.5 BAU/mL, while the highest was 9673.8 BAU/mL, with the mean concentration at 7535.8 BAU/mL. This average concentration was 2.8 times of the average antibody concentration derived from all participants post third dose.

For 27 individuals with breakthrough infections, the average antibody concentration prior to infection was 1911.3 BAU/mL, while the highest antibody concentration was 8717.7 BAU/mL. Since it is known that COVID-19 antigen rapid test has relatively poor clinical sensitivity and specificity when compared with PCR and WGS, the details of those 27 cases are not shown and further discussion focuses on the seven breakthrough cases that were characterized by WGS.

### 3.3. Individual Trend of Vaccine Mediated Serological Responese

**Figure 1:**
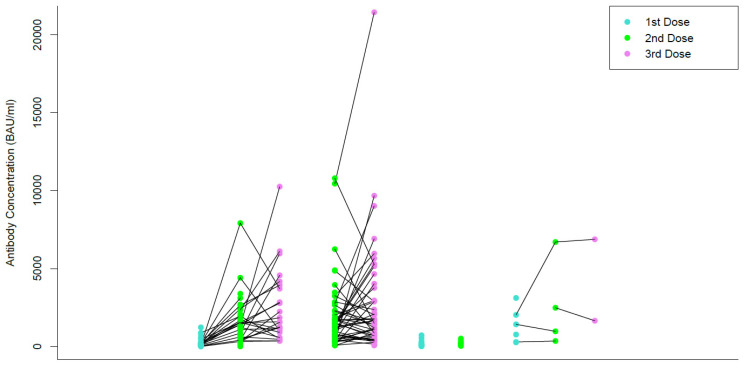
Antibody concentration after the first, second, and third doses.

Figure 1 shows the change in antibody levels after receiving the first, second, and third dose of each participants’ respective vaccines. Each line is drawn from the antibody levels measured after the first dose, to the second, and then third dose for the same participant. In Figure 1, the colour of the dot corresponds to the dose of the vaccine that a participant received. A blue dot represents the antibody concentration of a first dose, a green dot represents the antibody concentration of a second dose, and a pink dot represents the antibody concentration of a third dose. For all vaccine groups, 98.2% of participants demonstrated a higher second dose antibody concentration than the first dose, and 58.9% had a higher third dose antibody concentration compared to the second dose (i.e., 41.1% became lower on the third dose). In the BNT162b2 category, the median antibody concentration between the first and second dose increased by a factor of 10.3, and 1.2 between the second and third dose. It should be noted that decreases in individual antibody concentration were also observed between the second and third dose. 100% of participants who received the BNT162b2 vaccine demonstrated an increase in antibodies between the first and second dose, while only 66.6% of participants demonstrated an increase in antibodies between the second and third dose. In the Mixed Dose category, the median antibody concentration increased by a factor of 1.3 between the second and third dose. Additionally, only 54.2% of participants demonstrated an increase in antibody concentration, while 45.8% demonstrated a decrease. In the mRNA-1273 category, the average antibody concentration between the first and second dose increased by a factor of 1.6, and 2.4 between the second and third dose. 66.6% of participants demonstrated an increase in antibody concentration between the first and second dose, while only 50% of participants demonstrated an increase between the second and third dose. The cohort of AZD1222 vaccine had limited participants and no comparisons between doses could be made. Times of increase were not provided in the last two categories due to a limited number of participants. A Kruskal–Wallis test was conducted to compare the first (120.8 BAU/mL), second (1245.2 BAU/mL), and third dose antibody concentrations (1570.5 BAU/mL) of the BNT162b2 group, which demonstrated a significant difference in antibody concentration between each group (*p* < 0.001). A Kruskal- Wallis test was conducted to compare the second (1146.6 BAU/mL) and third dose (1559.1 BAU/mL) average antibody concentrations from the Mixed Dose category, which demonstrated no significant difference between the antibody concentrations (*p* = 0.15). Due to a limited number of participants, no statistical analyses were performed for the AZD1222 or mRNA-1273 groups.

**Figure 2:**
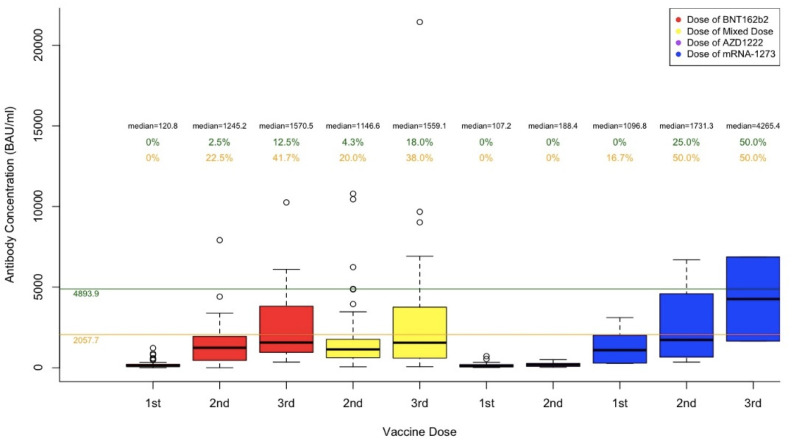
Serological Responses post 3 Doses and Comparison to Those in the Breakthrough Cases. The upper green line of 4893.9 BAU/mL is the highest antibody concentration prior to a known breakthrough infection. The lower orange line of 2057.7 BAU/mL is the average of antibody concentrations in seven infections confirmed by WGS. The percentages shown in green represent the percentage of antibody concentration that is above 4893.9 BAU/mL per each dose of each vaccine type, whereas the percentages in orange represent those above 2057.7 BAU/mL.

Figure 2 shows the median and the range of participant antibody concentrations after receiving the first, second, and third dose of their respective COVID-19 vaccine. In Figure 2, the colours of the boxplots correspond to the type of vaccine that they represent. Red corresponds to the antibody concentrations from participants who received BNT162b2 for their first, second or third dose. Yellow corresponds to the antibody concentrations from participants who received a Mixed dose for their second or third dose of the vaccine. Purple corresponds to the antibody concentrations from participants who received AZD-1222 for their first or second dose of the vaccine, and blue corresponds to the antibody concentrations from participants who received mRNA-1273 for their first, second or third dose. The boxplots are categorized by vaccine types, including BNT162b2, Mixed Dose, AZD1222, and mRNA-1273. When compared with 4893.9 BAU/mL (the highest antibody concentration prior to a known breakthrough infection), post second dose of vaccination, 2.5% of participants who received both BNT162b2, 4.3% of participants who received mixed vaccines, and 25.0% of participants who received both mRNA-1273, had antibody levels above 4893.9 BAU/mL. No participants that received the AZD1222 vaccine for both doses had antibody levels above 4893.9 BAU/mL post second dose. Following a third dose of vaccination, 12.5%, 18.0%, and 50% of participants who received either all doses of BNT162b2, mixed, and all doses of mRNA-1273, respectively, had antibody levels above 4894.9 BAU/mL. When all vaccine groups were combined, the percentage over 4893.9 BAU/mL was 0%, 4.0%, and 17.1% for the first, second, and third dose, respectively.

When compared with 2057.7 BAU/mL (the average antibody concentrations in seven infections confirmed by WGS), 22.5%, 20.0%, and 50.0%, of participants who received two doses of BNT162b2, two doses of mixed types, and two doses of mRNA-1273, respectively, had antibody levels above 2057.7 BAU/mL. Post third dose of vaccination, 41.7%, 38.0%, and 50.0% of participants who received all BNT162b2, mixed dose, and all mRNA-1273, respectively, had antibody levels above 2057.7 BAU/mL. When all vaccine groups were combined, the percentage over 2057.7 BAU/mL was 1.5%, 19.0%, and 37.2% for the first, second, and third dose, respectively.

A Kruskal–Wallis test demonstrated a significant difference in antibody concentration levels amongst the different vaccine types for the first and second dose (first dose, *p* < 0.002; second dose, *p* < 0.001). However, no significant difference was seen in antibody concentration levels amongst the different vaccine types for the third dose (*p* = 0.47).

## 4. Discussion

Our data demonstrates that when additional dosing of SARS-CoV-2 vaccines were administered in healthy individuals, the median antibody levels (for all vaccines combined) continuously rise from the first, second, to a booster dose (*p* < 0.002). This finding concurs with other observations, where similar increases were found [8,9,10,11]. There is evidence that SARS-CoV-2 vaccination induces mucosal antibody responses that correlate well with antibody concentration in the circulation, which supports the use of serum antibody level to evaluate immunity [12]. In our cohort, there was a 7.2-fold increase in antibody levels from the first to second dose, and a 1.5-fold increase from the second to third dose. When we categorized the antibody concentration into four groups, we observed a significant statistical difference among antibody concentration in the first (*p* < 0.002) and second dose (*p* < 0.001), but not in the third dose (*p* = 0.47). This suggests that the difference in vaccine-mediated serological response diminishes after multiple doses are administered. Our findings are in agreement with some observations [10] but contradict others [8]. As the unit of testing result are reported differently (EIA units in [10], U/mL in [8], and BAU/mL in our study), we suspect this difference may be due to different methods used to measure the antibody levels. This may reflect different serological assays used which render results incomparable. Standardized and comparable serological testing is essential to evaluate humoral immunity post vaccination. We suggest all methods should be traceable to the WHO International Standard for anti-SARS-CoV-2 immunoglobulin (NIBSC code 20/136) as is our method [13].

Interestingly, although on average there was 1.5 times increase in antibody levels from the second dose to the third, 41.1% of our participants demonstrated a lower third dose level when compared with the second. When serological responses are closely monitored at multiple shorter intervals longitudinally, it is known that the antibody levels peak at about 4 to 6 weeks, then gradually taper down over time [14,15]. In our cohort, the average interval between the third dose and blood collection was 4.5 months, while it was 2.4 months between blood collection and the second dose. This prolonged interval following the third dose was preselected intentionally to evaluate long-term immunity prior to the next booster dose with single blood collection. Likely due to this prolonged interval of 4.5 months, some individuals demonstrated the third dose antibody levels lower than that of their second dose. Additionally, we observed that the average of third dose antibody levels was 1.5 times that of the second dose, whereas other studies have reported approximately a 4 times difference. This is likely due to the shorter 4-week interval between blood collection and vaccination [10]. Our finding of lower antibody levels at approximately 4.5 months post a third dose in some individuals (when compared with the second dose) could inform the public health policy regarding the optimal vaccination interval. In Canada, for individuals 12 years of age and older for whom boosters are offered, the recommended interval is ≥6 months after completion of a primary series [16]. The FDA suggests that the vaccine may be administered to individuals 50 years of age and older at least 4 months after receiving a first booster dose [17]. While such recommendations are appropriate for the majority of our population, a small percentage of the population may benefit from shorter vaccination intervals to ensure that their antibody levels do not drop significantly. Clearly, there are multiple factors to be considered in the development of public health policy and maintaining a high antibody level is only one of those factors.

Another less likely explanation for the decreased third dose serological responses in some individuals is that they have reached the peak antibody concentration possible. Their lower antibody levels could be due to analytical variation in the serological method, i.e., their true antibody levels have peaked and only fluctuate slightly between the second and third doses. This hypothesis contradicts other findings which showed antibody responses continuously rise from the third to fourth dose [18]. Nevertheless, it is important to understand whether booster doses further increase serological responses or mostly only maintain existing antibody levels. We are currently following up our participants for the fourth dose antibody measurement.

The adaptive immunity includes humoral immunity, which protects against extracellular microbes and their toxins, and cell-mediated (or cellular) immunity, which is responsible for defense against intracellular microbes. Post-vaccination, it is known that in the absence of antibodies, CD8 + T lymphocytes specific to conserved viral epitopes correlated with cross-protection against symptomatic influenza [19]. A similar phenomenon is also seen in the case of rubella, where low antibody levels may not always be indicative of susceptibility to infection [20]. T lymphocytes comprise a major part of the adaptive immune response to the SARS-CoV-2 virus [21]. Understanding the T lymphocyte response to SARS-CoV-2 can increase our knowledge about the immunogenicity of the vaccines. The assessment of cellular responses relies on time-consuming, laborious, and expensive assays, and as such, are not routinely used. Therefore, serological testing is the primary tool to evaluate the efficacy of most vaccines.

For most other vaccines, a universal cut-off based on semi-quantitative or quantitative ELISA is often chosen to represent protection and immunity. This cut-off is in the range from 1 to 64 times that of the seroconversion concentration [4]. To date, no vaccine developed for other pathogens requires a serological response of more than 64 times of the seroconversion concentration to render immunity. The method manufacturer, Euroimmun, recommends a cutoff of 35.2 BAU/mL to indicate seroconversion (confirmed by our unpublished data). The median antibody level of 1046.4 BAU/mL post second dose is 29.7 times that of the seroconversion level, while the median antibody level of 1604.7 BAU/mL post third dose is 45.6 times that of the seroconversion level. This indicates that most vaccines approved are effective in producing robust antibody responses. Among 140 participants, 27 (19.3%) developed breakthrough infections, which were primarily identified by rapid antigen test. The average antibody concentration was 1911.3 BAU/mL prior to the infections in those 27 infections. Among the seven breakthrough cases which were further categorized by WGS, the average antibody concentration prior to infection was 2057.7 BAU/mL, while the highest was 4893.9 BAU/mL, representing 58.4 and 139.0 times that of the seroconversion level, respectively. This suggests that certain SARS-CoV-2 variants (Delta and especially Omicron) are more contagious than most other pathogens for which we have developed effective vaccines, likely due to their capacities to evade neutralization more efficiently [22].

Before the surge of various variants of concern, SARS-CoV-2 vaccine breakthrough infections occurred in only a small fraction of all vaccinated persons and accounted for a small percentage of all COVID-19 cases [23,24,25]. Prevention against Delta variant infection was reported at approximately 70% in recent literature [26,27]. Based on recent surveillance data, CDC has reported reduced protection against symptomatic infection at 62.8% for Omicron post 3 Janssen mRNA doses at 2 to 4 months since last dose [28]. Breakthrough is observed with other vaccines, such as the influenza vaccine [29]. Many factors likely contribute to the prevention of breakthrough infections, such as adaptive immunity in the host and public health measures. Our data, based on a small cohort, suggests that vaccine mediated antibody response is not the only factor contributing to the prevention of infection, as 62.8% of triple vaccinated and 81.0% of double vaccinated individuals have SARS-CoV-2 antibody responses lower than the average antibody concentration of known breakthrough cases. Therefore, effective public health measures (e.g., social distancing or masking) likely contributed to the observed 62.8% immunity for Omicron infection post third dose, in addition to antibody responses. Conceivably, a lower serological response is potentially protective against infection when the dose of viral exposure is low, which could be achieved by effective public measures. Our findings suggest that different from other vaccination programs, a universal cut-off based on serological response likely is not appropriate for SARS-CoV-2 vaccines as public health measures could further improve immunity for infection for individuals with low serological responses. A larger cohort is required to compare with our findings, which are based on limited participants and breakthrough infections, and therefore are not conclusive. While we acknowledge that the antibody responses in some individuals may not be sufficient to provide protection against infection, the critical role of vaccination in this pandemic could not be underestimated. To highlight this effect, the assessment of vaccine effectiveness should also focus on severe outcomes including hospitalization, ICU admission, or death, and not only breakthrough infections [30,31].

## 5. Limitations

First, our cohorts were small in size. Second, the antibody trend cannot be monitored with a single blood collection post each dose; however, this is the approach (using single serological testing) to evaluate other vaccinations. There were sizable variations in blood collection intervals post each dose, e.g., 2.4 months post the second dose versus 4.5 months post the third dose. Antibody measurements at strictly controlled time intervals would make statistical comparisons more reliable. In this manuscript, we did not test neutralising antibody concentration. Neutralising antibodies might represent the best method to evaluate humoral immunity, but their use for routine population-based testing is unpractical due to technical requirements [32], and they do not provide equal protection against all variants [33]. The focus on humoral immunity may not reflect long term immunity in the form of memory B cells or in the T-cell response. Studies to assess memory B cell function and T-cell immunity using assays are underway.

## 6. Conclusions

Most vaccines are effective in producing robust antibody responses when more than one dose is given, and the more doses the higher serological responses. Likely due to the highly contagious nature of SARS-CoV-2 variants, 37.2% of triple vaccinated, 19.0% of double vaccinated individuals, and 1.5% of individuals with a single dose have SARS-CoV-2 antibody responses higher than the average antibody concentration prior to known breakthrough cases. The lower antibody levels in many participants even after three doses suggest additional vaccination is likely required to ensure immunity.

## Figures and Tables

**Table 1:** Characteristics of 140 Healthy Study Participants.

	Characteristic	Antibody Concentration, BAU/mL, Median (IQR)
Age, median (range)	55 (20–89)	
Sex (n)		
Male (%)	46 (32.9)	
Female (%)	94 (67.1)	
Vaccine Received (n)		
**FIRST DOSE**		
BNT162b2	37	120.8 (81.9–216.0)
AZD1222	20	107.2 (55.1–192.5)
mRNA-1273	6	1096.8 (410.8–1877.8)
Median antibody concentration for all vaccines (n and median)	63	143.6 (79.0–266.6)
Days between 1st dose and blood collection, mean (SD)	62.8 (±28.4)	
Days between 1st and 2nd dose, mean (SD)	77.1 (±25.9)	
**SECOND DOSE**		
BNT162b2	40	1245.2 (475.2–1951.5)
Mixed	70	1146.6 (634.5–1760.1)
AZD1222	11	188.4 (88.6–279.1)
mRNA-1273	4	1731.3 (820.6–3541.2)
Median antibody concentration for all vaccines (n and median)	126	1046.4 (423.9–1738.2)
Days between 2nd dose and blood collection, mean (SD)	72.7 (±51.4)	
Days between 2nd and 3rd dose, mean (SD)	179.8 (±42.5)	
**THIRD DOSE**		
BNT162b2	24	1570.5 (985.7–3765.4)
Mixed	50	1559.1 (627.8–3561.3)
AZD1222	0	NA
mRNA-1273	2	4265.4 (2959.7–5571.2)
Median antibody concentration for all vaccines (n and median)	79	1604.7 (700.1–3764.0)
Days between 3rd dose and blood collection, mean (SD)	135.8 (±35.1)	
*p* value *	<0.001	

* *p* < 0.001 when comparing the antibody concentration between the median antibody concentration of the three doses.

**Table 2:** Characteristics of seven breakthrough cases confirmed by PCR and WGS.

Characteristic	Delta	Delta	Omicron	Omicron	Omicron	Omicron	Omicron
Age	58	58	57	70	49	56	45
Sex	Female	Male	Female	Male	Male	Female	Male
**FIRST DOSE**							
Vaccine	mRNA-1273	BNT162b2	BNT162b2	AZD1222	BNT162b2	AZD1222	BNT162b2
Days between blood collection and 1st dose	56 days	26 days	61 days	N/A	N/A	N/A	N/A
Antibody (BAU/mL)	268.5	538.2	118.2	N/A	N/A	N/A	N/A
Interval between 1st and 2nd dose	64 days	49 days	80 days	66 days	114 days	79 days	91 days
**SECOND DOSE**							
Vaccine	mRNA-1273	BNT162b2	mRNA-1273	BNT162b2	BNT162b2	BNT162b2	BNT162b2
Interval between blood collection and 2nd dose	54 days	25 days	25 days	107 days	115 days	67 days	56 days
Interval between 2nd dose blood collection and infection	102 days	115 days	163 days	85 days	29 days	252 days	352 days
Antibody prior to infection (BAU/mL)	3249.4	2748.4	4893.9	209.1	231.5	725.7	2346.6
Interval between 2nd and 3rd dose	N/A	N/A	177 days	N/A	N/A	N/A	170 days
**THIRD DOSE**							
Vaccine	BNT162b2	BNT162b2	BNT162b2	BNT162b2	BNT162b2	BNT162b2	BNT162b2
Interval between blood collection and 3rd dose	N/A	N/A	107 days	N/A	N/A	N/A	202 days
3rd dose Antibody Result (BAU/mL)	5962.5	9019.5	N/A	N/A	9673.8	6915.3	6108

## Data Availability

The data presented in this study are available on request from the corresponding author.
